# Computational modeling of maxillary canine orthodontic movement

**DOI:** 10.1016/j.heliyon.2024.e34175

**Published:** 2024-07-05

**Authors:** Shai Yona, Oded Medina, Nir Shvalb, Rachel Sarig

**Affiliations:** a The Department of Mechanical Engineering, Faculty of Engineering, Ariel University, Israel; b The Goldschleger School of Dental Medicine, Faculty of Medical and Health Sciences, Tel Aviv University, Tel Aviv, 6997801, Israel; c Dan David Center for Human Evolution and Biohistory Research, Faculty of Medical and Health Sciences, Tel Aviv University, Tel Aviv, 6997801, Israel

**Keywords:** PDL, Simulation, Stresses, Impacted canine, Orthodontic tooth movement

## Abstract

**Objectives:**

The current study aims to explore the stress distribution along the roots of palatally positioned maxillary canines during orthodontic movement using a novel computational spring model.

**Methods:**

An experimental analysis based on the spring-model was utilized to calculate Orthodontic Tooth Movement (OTM) and the resulting stresses. Two sets of experiments were conducted: the first set compared stresses on a canine resulting from a single force and a force-couple, while the second set simulated canines' traction during instantaneous movement with varying original tooth angulations using different off-the-shelf orthodontic coils. In total, 130 simulations were performed.

**Results:**

The model provided estimated stress distribution throughout the OTM with the expected movements, producing consistent outcomes with prior findings. In the first set of experiments, the force couple exhibited an average stress of 43 KPa, while a single force yielded 51 KPa on average. The maximum stress observed was 63 KPa for the force couple and 130 KPa for a single force. Note that the stress distribution attributed to the force couple was alleviated in comparison to the stress distribution caused by a single force. Force couples generated higher average stress. In the second experiment, the application of an occlusally-directed inclined force led to reduced stress levels overall. For instance, when a 200 g distal force was exerted on the canine, it generated an average stress of 20 KPa, whereas applying a force of the same magnitude in an occlusal-distal direction resulted in a lower average stress of 15.5 KPa.

**Conclusions:**

Lower average stress levels when using a force couple indicate that larger loads might be safely applied for rotational movements. Given that areas under maximal stress are prone to damage, orthodontic treatment planning should carefully consider stress distribution to minimize potential harm in these high-stress zones. The results also suggest that force couples enable the use of stronger forces than a single force. Additionally, it is advisable to extrude the tooth initially before starting any horizontal movement towards the target position.

**Clinical significance:**

Given that orthodontic treatment often relies on virtual planning, incorporating a variety of methods to evaluate stress distribution within the treatment strategy could offer numerous benefits. Such an approach holds the potential to improve both the efficiency and safety of orthodontic treatments, especially in complex cases that require the application of high forces.

## 1 Introduction

Orthodontic tooth movement (OTM) is initiated and controlled by mechanical stimuli, generated by forces applied on the crown of the tooth [1]. The forces are partially governed by the periodontal ligament (PDL), which connects the teeth to the surrounding alveolar bone. Examining the stress-strain responses of teeth, alveolar bone, and PDL to orthodontic loading is of importance to apply optimal forces during treatment. The PDL width ranges from 0.15 to 0.38mm [2,3] and a range of the PDL modulus of elasticity is considered in the literature, most studies specify values of 600–900 Kpa [[4], [5], [6], [7], [8], [9]], or 1180 Kpa [10] The PDL has viscoelastic, inhomogeneous, anisotropic, and nonlinear properties [11], however, it is commonly approximated to have isotropic behavior [4,10,12,13]. We shall follow this convention here regarding its' elasticity properties.

Recommended force magnitudes for various treatments were tested and produced satisfying treatment results are available in the literature [14]. Lee [15] provided a recommended range for the optimum horizontal force to be applied on a canine, which is between 150−260gf and a recommended range for the applied stress of $165‐185\text{gf}/cm^{2}$, or slightly higher. Later on, the same author [16] suggested an average stress of $197\text{gf}/cm^{2}$ where a minimal 1500μ strain is required for remodeling process to take place [11,17]. Yet, one should note that in-vivo measuring of the pressure or strain in the tissues involving tooth movements is still out of hand [2].

Most of the available mechanical theoretic research on OTM rely on the finite element method (FEM), (c.f., [5,10,13,18]). However, in most cases the FEM analysis did not consider the remodeling process and extracted the stress and strain distributions due to instantaneous movement, this cannot provide full indication on the actual tooth movement, since the latter is governed by remodeling process which will be clarified below. The biological process of orthodontic tooth movement includes three stages [19]: (1) Tooth movement within the dental alveolus with no substantial deformation upon the bone; (2) A biological process during in which no movement takes place. This stage is triggered when a sufficient force is exerted on the tooth and completed after a sufficient time period. Once this stage is completed; (3) the last stage of alveolar bone remodeling begins, where the resorption and formation processes are applied depending on the nature of stresses exerted upon the PDL (i.e., compression and tension respectively). However, the resorption process is faster than formation [20].

Several studies addressed the entire OTM process, including the remodeling stage, some used FEM approach [[21], [22], [23], [24]]. Certain studies required re-meshing the model whenever reaching an intermediate equilibrium, such an approach demands large computational resources and is time consuming [25]. In 2016 Hasegawa et al. [25] used an image-based voxel level set in their iterative procedure to overcome the large computation problem.

**Fig. 1:**
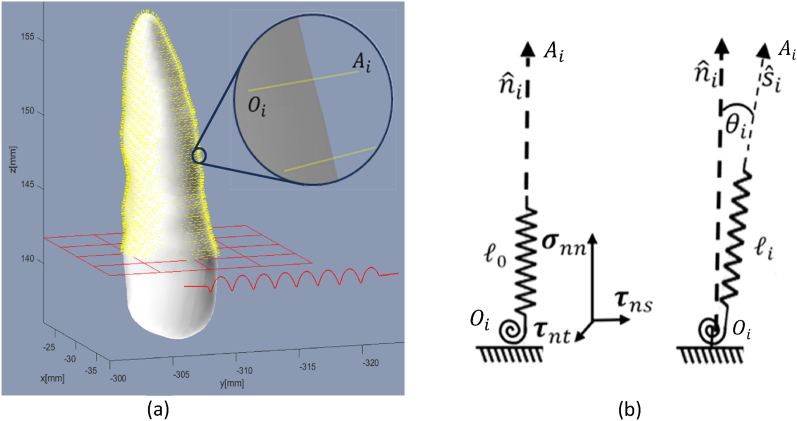
PDL model: (a) Depicts a right maxillary canine with springs (colored in yellow) representing the relaxed state of the PDL before the initiation of orthodontic force (presented as red helix), the gingival line is outlined by a red mesh. (b) Depicts a single spring-block occupying a segment of the tooth surface (lower end) and anchored to the alveolar bone (upper end). Each block is comprised paired linear and torsional springs ((b) left). These springs undergo both linear $l_{i}-l_{0}$ and shear $\tan\left(\theta_{i}\right)$ deformations (right), where $l_{0}$ denotes the spring's initial length. $O_{i}$ and $A_{i}$ denotes the spring's edges that are connected to the tooth and the alveolar bone edges, respectively. (For interpretation of the references to color in this figure legend, the reader is referred to the Web version of this article.)

In a previous study [26] the authors introduced a model aimed to describe the full sequence of OTM. We established a springs-model to calculate the intermediate stress distribution and the corresponding tooth movement. The model takes into account the remodeling process at which the aforementioned stresses are relaxed. We considered that tooth movement is an instant occurrence while remodeling – the formation and resorption processes of the tissue require longer periods of time. These two steps are repeated until equilibrium is obtained. We shall briefly review the model details:

(1) The numerical model assumes the PDL to be isotropic with a Poisson ratio of ν=0.45 and the spatial shape of the considered tooth to be known (via a CBCT scan, say). We followed literature data for the PDL properties and determined young's modulus of 0.68 MPa and thickness of 0.265 mm [6].
(2) The Instantaneous behavior of the PDL is modeled as a set of springs connected to tooth-face and alveolar bone. The governing idea is that tissue response under orthodontic force [2] reduces the total energy of the system (i.e., tissue and orthodontic appliances overall energy). We represented the microstructure of the PDL as pairs of springs (depicted in Fig. 1 a,b): a linear spring and a shear spring (aligned tangent to the tooth facet) (Fig. 1 b). In a relaxed state, the linear springs are perpendicular to the tooth (Fig. 1 a). The stiffness of each spring was normalized with respect to the area it occupies. The PDL tissue can undergo compression up to a given limit where the resistance of the tissue prevents further elastic movement. We model this by applying a minimal length constraint of spring's projection on the normal to the tooth face.

During the energy reduction process, springs that exceed the edge of the surrounding tissue (the gingival line) due to tooth movement are removed. This occurs in real life for example when a tooth is extracted, damaging the PDL fibers.

(3) Remodeling process yields relaxation of the PDL, where a spring subjected to a given stress in a certain direction will result with movement of the anchorage point at rate which depends on the exerted stress.

In Fig. 1a, the model's configuration is illustrated, showcasing a tooth, an orthodontic force symbolized by a red coil, a gingival line depicted as a red mesh (which can be set to any orientation and position), and the springs representing the PDL tissue visualized by yellow lines. In Fig. 1b we illustrate the alteration in the state of an individual spring caused by tooth movement, which affects point $A_{i}$. However, when the remodeling process takes place, the point $O_{i}$ undergoes apposition, resulting in the relaxation of the spring.

**Fig. 2:**
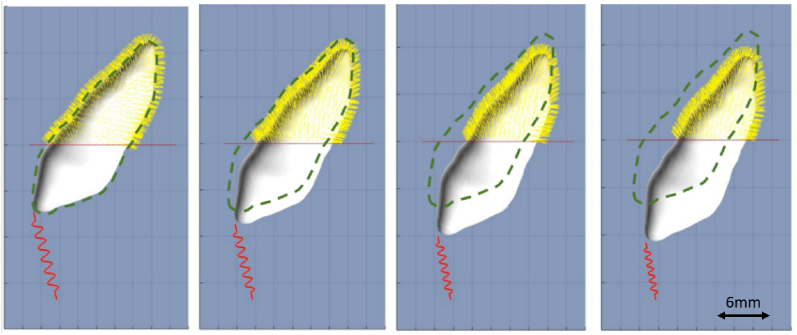
Tooth movement with remodeling. For display purposes the PDL springs (yellow) lengths are exaggerated. The initial position of the tooth is overlayed in green dashline. Orthodontic appliance displayed as red coil and gingival line is depicted as a red horizontal line. (For interpretation of the references to color in this figure legend, the reader is referred to the Web version of this article.)

Fig. 2 illustrates an example of maxillary canine extrusion, for display purpose the length of the PDL springs (yellow) is exaggerated. Orthodontic appliance is displayed as a red coil and gingival line is displayed as a horizontal red line. The initial position of the tooth is depicted as a green dashed line. Note the change in coil length and coil-tooth anchorage position as the tooth moves. The (yellow) PDL springs are removed when exceeding gingival line resulting in reduction of the overall coverage of the tooth.

The primary objective of this research is to examine the varied patterns of stress distribution along the roots in distinct orthodontic scenarios, particularly emphasizing cases involving palatally displaced canines that necessitate movement into the dental arch. This study is dedicated to an in-depth investigation of the effects of force direction and tooth angulation on root stress during orthodontic procedures. Through a methodical evaluation of the influence of different force vectors and tooth orientations on stress dispersion, this research strives to furnish a comprehensive understanding of the mechanical dynamics involved in orthodontic interventions for such clinical situations. The interplay between tooth orientation and the direction and magnitude of applied forces significantly impacts stress distribution along the tooth root. This study aims not only to elucidate these correlations but also to develop a predictive model to enhance understanding of the potential outcomes of orthodontic movements.

## 2 Methods

The computational model was implemented in a Matlab software on a 32 GB RAM Intel Core i7_9700 PC. A 3D model of the tooth was extracted from a CBCT scan of a healthy 14 years old male patient with palatally impacted canine.

Since a clinical direct stress measurement is not in hand one remains with the theoretic option. To better understand the resulting stresses when external loads are applied, we conducted a large set of experiments.

**Fig. 3:**
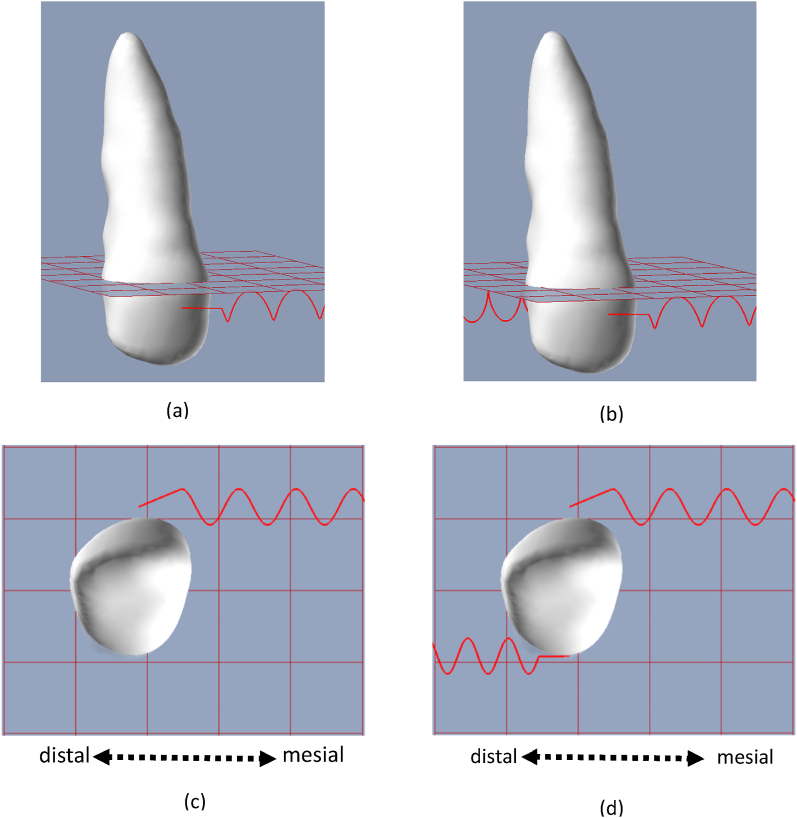
Configurations of a single mesial force (a,c) and a mesial-distal force couple (b, d) applied over an upper-right canine. These configurations were set to study the stress distribution over the PDL throughout OTM under single-force and a force-couple loadings. c, d present an occlusal view.

In the first set of experiments, we compared stress distributions when applying a force couple compared to the case where a single force was applied. Fig. 3a-d displays the configuration setting of the first experimental simulation; a single 2.94 N (300gf) mesial force connected at mid-point of the buccal surface (Fig. 3 a, c). The second configuration is based on a force couple (Fig. 3 b, d) of 2.94 N × 5.72 mm positioned coronally to gingival line; a mesial force of 2.94 N located in the mid-point of the buccal surface and additional distal force of 2.94 N connected at the mid-point of palatal surface.

**Fig. 4:**
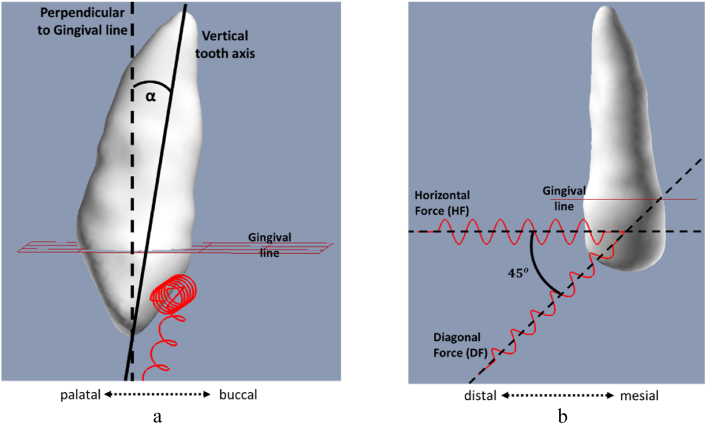
Tooth-load configurations for an upper-right canine, applied in the second simulation set. The orthodontic appliances are depicted as red coils. A single distal horizontal force (HF) or diagonal force (DF) was applied. The gingival line is displayed in red, and the orientation of the tooth denoted by α is determined by the angle between the perpendicular to gingival line and the vertical tooth axis. (For interpretation of the references to color in this figure legend, the reader is referred to the Web version of this article.)

In the second set of experiments, a model of a partially erupted maxillary canine was subjected to a wide range of forces in a set of scenarios. The parameter set included: (1) various tooth orientations represented by an angle (α) of $15^{\circ }$, ${\;0}^{\circ }$, $-15^{\circ }$ and $-30^{\circ }$ between the perpendicular to gingival line and the tooth main axis (Fig. 4a). (2) Two force directions were evaluated: from the mid-point of the buccal surface of the canine crown, a single force was applied either horizontal towards distal direction (HF) or towards distal-occlusal direction (DF) (i.e., diagonally $45^{\circ }$ to the horizontal direction) as depicted in Fig. 4b. (3) Properties of several of-the-shelf coils used in orthodontics. The simulation was provided with the values listed in columns (Fig. 7, Fig. 8): “Appliance model”, “Appliance K″ and “Appliance length”. “Appliance model” specifies the model of orthodontic wire denoting the rest length [mm] together with the stiffness type (L-light, m-medium H-Heavy). “Appliance K″ column specifies the stiffness coefficient of the coil and the “Appliance length” column specifies its initial loaded length (activation length). The properties of the Appliance model and Appliance K followed the values provided by the International Orthodontic Services (IOS) [27]. In this set of experiments, we also present the stresses under exceedingly high forces (e.g > 400gf). This may result in tissue damage and calcification that is not considered here in the remodeling process [26], thus stresses were recorded at the conclusion of the instantaneous movement.

The “Initial Force” column was calculated by Hook's law:

$$F_{i}=k\left(l_{l}-l_{r}\right)$$

where k is stiffness coefficient of the coil, $l_{l}$ is the activation length of the coil and $l_{r}$ is its rest length.

The governing stress over the PDL tissue we used here is the “equivalent” Von-Mises stress:

Equation 1 $$\sigma_{\max}=\sqrt{\sigma^{2}+3\tau^{2}}$$

which is a function of the normal stresses σ and shear stress τ. It should be noted, though, that literature behind Von-Misses stress is based on the premise that a material fails by shear strain, which as far as the authors' knowledge, had not been examined yet for the PDL tissue but is widely assumed in literature (e.g. Ref. [10]).

## 3 Results

**Fig. 5:**
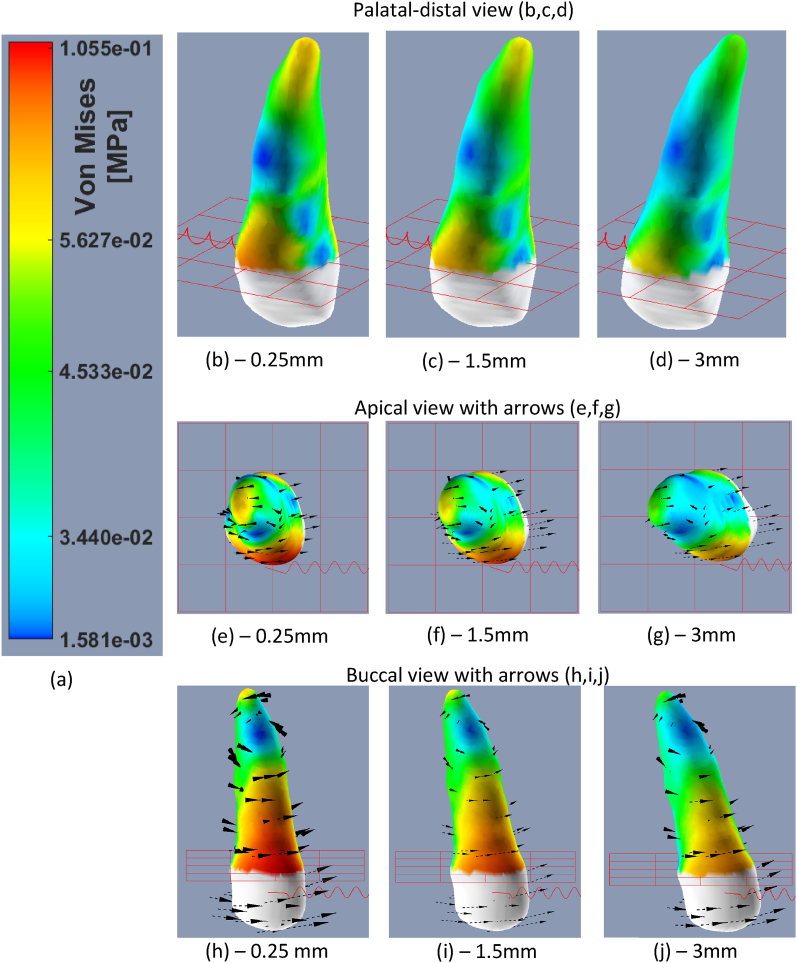
Von Misses stress distribution over upper-right canine PDL tissue where a single 300gf mesial force was applied. The stress values are specified in the color bar (a). (b), (c),(d) a palatal-distal view at 0.25 mm, 1.5 mm and 3 mm anchorage movement. The black arrows display the direction of movement of each area in the tooth. (e), (f), (g) an apical view at 0.25, 1.5 and 3 mm movement. (h),(i),(j) a buccal view at 0.25,1.5 and 3 mm movement, note that the black arrows imply a tipping movement. (For interpretation of the references to color in this figure legend, the reader is referred to the Web version of this article.)

**Fig. 6:**
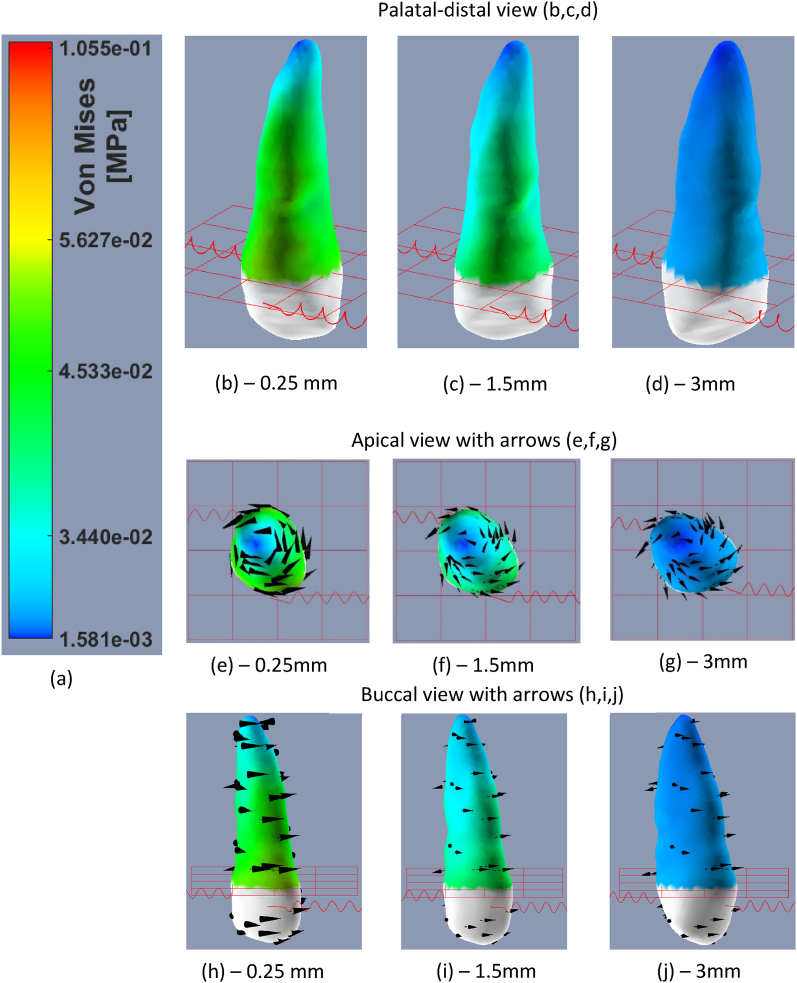
Von Misses stress distribution over upper-right canine PDL tissue where a couple 300gf forces was applied. The stress values are specified in the color bar (a). (b),(c),(d) a palatal-distal view at 0.25 mm, 1.5 mm and 3 mm anchorage movement. (e), (f), (g) an apical view at 0.25, 1.5 and 3 mm movement. The black arrows display the direction of movement of each area in the tooth, implying a rotational movement. (h),(i),(j) buccal view at 0.25,1.5 and 3 mm movement. (For interpretation of the references to color in this figure legend, the reader is referred to the Web version of this article.)

Fig. 5(a–j) display the stresses over the PDL and the OTM due to a single force where Fig. 6(a–j) display the stresses and OTM due to a force couple. In general, the deviation of the stresses due to a force couple was reduced. The force couple resulted in a lower maximum stress (59 KPa compared to 80 KPa on instantaneous movement and 63 KPa compared to 130 KPa on overall max).

**Fig. 7:**
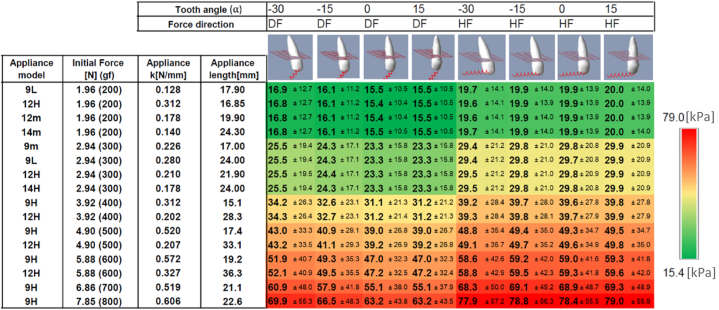
- The PDL's mean Von Misses stresses and their standard deviations (small fonts) given in [kPa], for various of-the-shelf orthodontic coils (see IOS [27]). The appliance model specifies the rest length of the coils in [mm] and the colors correspond to the stress values as in the sidebar. (For interpretation of the references to color in this figure legend, the reader is referred to the Web version of this article.)

**Fig. 8:**
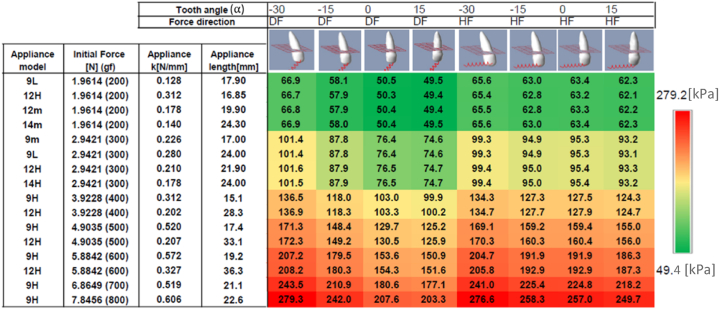
Maximal Von Misses stress calculated over the PDL tissue given in [kPa].

As the average stress of the force couple was higher at the end of instantaneous movement (40 KPa compared to 31 KPa), after 0.25 mm of anchorage movement (where the average force was about at it maximum) the force couple produced a lower average stress: 43 KPa compared to 51 KPa. The single force produced higher stresses in the areas close to the gingival line in the buccal side (Fig. 5 b,e,f), while the force couple generated higher stresses in the palatal and buccal surfaces near the gingival line lower stresses in the apex (Fig. 6 b,e,h). A tipping movement was observed due to the single force (see arrows in Fig. 5j) and rotation movement was observed due to the force couple (arrows in Fig. 6g).

At the second set of experiments the mean and the maximum values of the entire stress distribution exerted on the PDL were calculated at the end of the instantaneous movement. Fig. 7 display the mean stress values and the corresponding standard deviation over the PDL surface (provided in small fonts). This is demonstrated in scenarios of various tooth angles and force directions over different orthodontic coils. We simulated of-the-shelf coils with varying properties (length and stiffness) as published by IOS [27].

For display purposes the results in Fig. 7, Fig. 8 were colored, the minimum value is displayed in green and the maximum in red (provided in the color bar). The colors correspond to the stress range provided in the sidebar in the figure.

Fig. 8 display the maximum stress over PDL at the same set of scenarios as in Fig. 7.

## 4 Discussion

In this paper, we present a new methodology for computing tooth displacement throughout the entire process of orthodontic tooth movement, as well as the corresponding stresses exerted on the PDL. The model assumes that the following are available: 1. The spatial shape of the tooth, 2. The properties of the PDL (thickness and modulus of elasticity) and 3. Position and modulus of elasticity of the involved orthodontic appliances.

In our previous publication where the spring model was first introduced [26], we extensively detailed and comprehensively compared it to other existing models. The spring model was tailored to emulate the characteristics of the PDL tissue, resulting in reduced complexity and greater flexibility compared to conventional finite element methods. Consequently, it demands fewer computational resources. This model provides the trajectory of the tooth throughout the treatment and the stress distribution over the PDL tissue throughout OTM. In all of the clinical cases detailed in Ref. [26], the model's results demonstrated accuracies below 0.3 mm and 2°. Furthermore, it is applicable for multiple orthodontic forces and sequential multi-stage treatments, enabling the activation and reconfiguration of orthodontic forces.

The first set of experiments suggests that applying a horizontal force (either single or couple) to a single-rooted tooth generates higher stresses near the alveolar crest and, to a lesser extent, at the apex (see Fig. 5). The observed distribution pattern is likely attributed to the configuration of the PDL springs, as greater distance from the tooth's center of rotation causes larger deformation of the springs, which is consistent with findings from previous studies [10,13]. Furthermore, the average Von Mises stress on a force-couple was found to be greater during instantaneous movement. This is likely attributed to the significant impact of shear stresses to the equivalent Von Mises stresses., which are larger when a force-couple is applied (as per equation (1)). However, the maximum stress observed was lower and the relaxation rate observed due to the remodeling process was higher. Thus, if possible, clinicians may consider utilizing a force-couple to decrease the maximum stress level.

The second series of experimental simulations consisted of 128 scenarios in which tooth orientation and force direction were evaluated under several properties (stiffness and activation length) of actual orthodontic appliances. Tooth orientation was evaluated at angles of −30°, −15°, 0°, and 15°, with initial force ranging from 1.96 N (200 gf) to 7.84 N (800 gf). The magnitude of the applied force had the greatest effect on the average stress, with only minor differences found due to other configurations. However, the maximum stress (Fig. 8) revealed significant differences, as a vertically oriented tooth resulted in lower stress compared to an acute angle, which produced a larger maximum stress.

The direction of the force applied affects the distribution of stress, with horizontal force (HF) resulting in higher mean stress compared to diagonal force (DF), regardless of tooth orientation. However, the difference between the HF and DF maximal stress values was mainly observed in cases of vertically oriented tooth (α = 15). These findings suggest that when the force is directed towards the vertical axis of the tooth (DF), lower stress is generated, while a horizontal force (HF) results in a higher stress distribution. The standard deviation values were found to be approximately 70 % of the mean stress. While the highest stress values were observed in the alveolar crest, some significant stress values were also found in the apex. These findings suggest that clinicians should exercise caution since certain areas may experience stress levels that are significantly higher than the mean stress. It was suggested that the recommended optimal mean stress for OTM is $197\text{gf}/cm^{2}$ [16] (19.3 KPa). The configuration displayed in Fig. 7 at the upper-right side (i.e., HF, vertical tooth) produced mean stress of 20.0 KPa which is close to the recommended mean optimal stress. The set of force values that were analyzed in this study suggested that a force around 200gf should be considered as an optimal horizontal force for a vertical canine tooth. So, when force direction tends toward extrusion (Figs. 7 and 8; columns 3 and 4), both mean and maximal stresses decreased, which may allow for the use of an increased force to produce the optimal stress.

In the current study, we analyzed various configurations that may result in the suggested optimal stress. The rest length and stiffness of the coils are determined by the properties of the coil, while the amount of activation is determined by the clinician. In general, the configurations with a given initial force value (e.g. 200gf) yielded comparable results across the different scenarios. Our findings suggest that stress values are primarily influenced by the applied force, with higher forces leading to greater stress, while variations in length and stiffness have a minor impact. The results also demonstrated that as the mean stress rises, the standard deviation also tends to increase, highlighting the need for caution when working with heavy forces. However, it should be noted that we did not evaluate the effects of gradual changes in the stiffness coefficient of the coils throughout the treatment in our current study.

The configuration shown in the upper-right subfigure of Fig. 7 (HF, vertical tooth) resulted in a mean stress of 20.0 KPa. This is consistent with the findings of B. Lee [16], who suggested a mean pressure of 19.3 KPa (197 $\text{gf}/{\text{cm}}^{2})$ to determine the optimum force. Therefore, a force of approximately 200gf may be a suitable value for this configuration. When the force direction tends towards extrusion in the tooth vertical axis, both the mean and maximal stresses decrease, making it possible to use increased force to achieve faster movement.

### 4.1 Study limitations and future work

It is important to note that when extreme forces are applied, the tissue may respond with hyalinization. This response could potentially delay in movement and affect the stresses applied on the PDL. Currently, this phenomenon is not considered in this study, and it may have implications for very large forces (i.e., 400 gf or larger) after biological processes are initiated.

A common assumption in research is that the PDL tissue exhibits linear and isotropic mechanical properties [10,12,13]. However, this assumption is recognized as an approximation [11] which may affect the results, specially where the remodeling process takes place. Consequently, accounting for the precise elastic behavior of the PDL could potentially enhance the accuracy of the results obtained [11]. Similarly, the precision of the model can be improved by capturing the non-uniform PDL thicknesses by using improved CBCT future scans coupled with the Hounsfield scale [28,29].

The model utilized in this study provides a solution for the case where a single tooth, distant from other teeth, is being loaded. While this is a first approximation to the common situation where adjacent teeth interact. For the sake of simplicity, we assumed that the properties of the orthodontic appliances were fixed throughout the orthodontic tooth movement. Nonetheless, we acknowledge that the mechanical properties of the appliances may alter during OTM. Therefore, the model was designed to readily accommodate these potential alterations.

While assuming a flat formation for the gingival line, it is important to note that utilizing precise input data can significantly improve the accuracy of the model. For example, incorporating the use of intra-oral scanners can provide additional visual data, such as the accurate formation of the gums surrounding the tooth. It should be noted that the novel model utilized in this study does not provide information on the temporal duration of each gradient flow iteration, which means that the model does not provide a proper time scale per movement. It is important to consider the timeframe of the movement and addressing it in future studies may prove beneficial.

In a clinical context, the integration of diverse methodologies to evaluate stress distribution into orthodontic treatment plans can offer numerous benefits, given the prevalent reliance on virtual design. This comprehensive approach holds the potential to significantly improve the precision, effectiveness, and safety of orthodontic interventions, especially in complex cases that require the application of substantial forces, such as the repositioning of impacted canines within the dental arch.

## 5 Conclusions

Utilizing the digital model to conduct simulative experiments, we demonstrated the stress responses in the PDL tissue of a maxillary canine across various scenarios caused by instantaneous movement. The stress peaks and the variability in stress distribution across the PDL resulting from a force couple are comparatively lower than those caused by a single force. This suggests that when using a force couple, one can potentially increase the applied force. Furthermore, the simulative experiments also demonstrated that applying horizontal force induces greater stress levels in comparison to an inclined occlusal force. The findings are consistent with the clinical experience of orthodontics, indicating that this model can contribute to the understanding of clinical outcomes and processes.

## Data availability

The datasets generated and/or analyzed during the current study are available from the corresponding author on reasonable request.

## CRediT authorship contribution statement

**Shai Yona:** Writing – review & editing, Writing – original draft, Methodology, Formal analysis, Data curation, Conceptualization. **Oded Medina:** Writing – review & editing, Writing – original draft, Supervision, Methodology, Formal analysis, Conceptualization. **Nir Shvalb:** Writing – review & editing, Writing – original draft, Supervision, Resources, Methodology, Formal analysis, Conceptualization. **Rachel Sarig:** Writing – review & editing, Writing – original draft, Supervision, Methodology, Investigation, Conceptualization.

## Declaration of competing interest

The authors declare that they have no known competing financial interests or personal relationships that could have appeared to influence the work reported in this paper.
